# Supplementary material for: A machine-learning based objective measure for ALS disease severity
Source: NPJ Digit Med. 2022 Apr 8;5:45. doi: 10.1038/s41746-022-00588-8 (PMC8993812; doi:10.1038/s41746-022-00588-8)
Supplement: Supplementary file 1 — Supplemental Material [file 41746_2022_588_MOESM1_ESM.docx]

**Supplementary Materials**

**Exercise movements video**

For the accelerometer recordings, the set of movements/exercises prescribed to participants is included as a link to a video (approximately 10 minutes in duration):

<https://www.youtube.com/watch?v=4G5JaRHxFYM&t=1s>

**Predicted vs. Groundtruth ALSFRS-R Confusion Matrices.**

Supplementary Table 1A shows the confusion matrix of the voice ALSFRS-R values using the voice-based model predicting the speech FRS score. The model has an overall accuracy of 73% (AUC of 0.86). On recordings, with ALSFRS-R scores of 4 (normal), the model predicts them accurately on 620 of the samples’ 692 (90%) samples. On samples with scores 3 and 2, the model predicts them with an accuracy of 57% (213 of 315, and 80 of 141 respectively). The model overestimates a rating of 3 and predicts those as a 4 (29% cases), and in the case of a 2, it confuses it as a 3 or 1 in 23% and 14% of the cases, respectively. It predicts 40% of the recordings rated as 1 accurately and tends to confuse them mostly as a 0 (29%) and at other times as a 2 or a 4.It predicts 65% of the recordings with a score of 0 as 0, and the remaining as 1. In all cases, the model’s misclassifications appear reasonable since the range of variation is plus/minus 1.

Supplementary Table 1B shows the confusion matrix of the voice ALSFRS-R values using the accelerometer model. It presents the average predictions (rounded to the nearest integer) across ALSFRS-R scores pertaining to the six limb related functions (handwriting, cutting_food, dressing_hygiene, turning_in_bed, walking, climbing_stairs). As we can observe, the accelerometer model tends to overestimate the FRS score, providing a higher score if the participants rated themselves as 0,1,2 or 3. (i.e., it predicts these as 1,2,3 or 4 respectively just as often as it predicts the actual value). Overall, in 76% of the cases, the accelerometer predicts the same score or a score higher by 1 point. Performance of different accelerometer models Supplementary Table 2 shows the performance (AUC values) of all the accelerometer model variants described in the methods section on predicting the ALSFRS-R scores on the limb related functions.

**Performance on each of the test participants in the edaravone study.**

Supplementary Figures 3 and 4 present the groundtruth and predicted labels for each of the 54 participants in the subset of test participants in the Edaravone study. Supplementary Figure 3 shows the groundtruth speech ALSFRS-R values and predictions from the voice model, it also presents the slopes before and after (i.e. pre- and post-) beginning edaravone treatment. Supplementary Figure 4 is an analog of Supplementary Figure 3 on the accelerometer model. It presents the sum of groundtruth ALSFRS-R values on the six limb-related functions and predictions from the accelerometer model along with the slopes before and after beginning edaravone treatment.

**Supplementary Tables**

| Speech | Predicted Scores | | | | | | |
| --- | --- | --- | --- | --- | --- | --- | --- |
|  |  | 0 | 1 | 2 | 3 | 4 | Total |
| Ground-truth scores | 0 | 13 | 7 | 0 | 0 | 0 | 20 |
|  | 1 | 29 | 41 | 14 | 5 | 14 | 103 |
|  | 2 | 5 | 20 | 80 | 33 | 3 | 141 |
|  | 3 | 6 | 12 | 35 | 213 | 110 | 376 |
|  | 4 | 8 | 0 | 6 | 58 | 620 | 692 |

**Supplementary Table 1 A.** Voice prediction model confusion matrix indicating the number of predictions that replicate groundtruth self-reportedl ALSFRS-R scores for speech.

| Speech | Predicted Scores | | | | | | |
| --- | --- | --- | --- | --- | --- | --- | --- |
|  |  | 0 | 1 | 2 | 3 | 4 | Total |
| Ground-truth scores | 0 | 18 | 120 | 12 | 0 | 0 | 150 |
|  | 1 | 9 | 162 | 102 | 40 | 5 | 318 |
|  | 2 | 27 | 166 | 319 | 484 | 291 | 1287 |
|  | 3 | 8 | 8 | 64 | 412 | 504 | 996 |
|  | 4 | 0 | 0 | 21 | 183 | 578 | 782 |

**Supplementary Table 1 B.** FFT Multilabel MLP model. Confusion matrix of ground-truth ALSFRS-R scores with predicted ALSFRS-R scores on the sum of the scores from the six limb related functions (handwriting, cutting_food, dressing_hygiene, turning_in_bed, walking, climbing_stairs) [Sum of limb score predictions (argmax over the sum of the limb frs prediction distributions), Groundtruth is average of the sum of limb scores and rounded to the nearest integer.]

| Accelerometer Data | Model | climbing_stairs | cutting_food | handwriting | turning_in_bed | walking |
| --- | --- | --- | --- | --- | --- | --- |
| TBVM | Logistic Reg. | 0.627 | 0.564 | 0.508 | 0.617 | 0.564 |
| Raw 30Hz | small CNN | 0.70 | 0.68 | 0.60 | 0.77 | 0.75 |
| Uniform 1Hz | Logistic Reg. | 0.634 | 0.593 | 0.537 | 0.602 | 0.586 |
| Uniform 1Hz | MLP (multi-label) | 0.738 | 0.726 | 0.634 | 0.753 | 0.755 |
| FFT 1Hz | MLP (multi-label) | 0.701 | 0.733 | 0.645 | 0.756 | 0.756 |

**Supplementary Table 2.** FFT Multilabel MLP model. Confusion matrix of ground-truth ALSFRS-R scores with predicted ALSFRS-R scores on the sum of the scores from the six limb related functions (handwriting, cutting_food, dressing_hygiene, turning_in_bed, walking, climbing_stairs) [Sum of limb score predictions (argmax over the sum of the limb frs prediction distributions), Groundtruth is average of the sum of limb scores and rounded to the nearest integer.]

**Supplementary Figures**

**
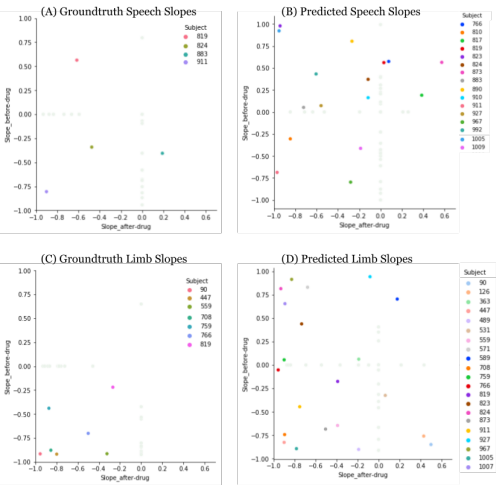
**

**Supplementary Figure 1.** Plots comparing slopes (r-values) on the subset of 54 test participants in the Edaravone study. The plots depict slopes (r-values) computed before (pre-Edaravone) and after (post-Edaravone) starting drug using the ALSFRS-R scores: (A) slopes from groundtruth speech scores, (B) slopes from speech scores as predicted by the voice model, (C) slopes from groundtruth limb scores [scores averaged over 6 limb-based movements], and (D) slopes from averaged limb-movement scores as predicted by the accelerometer model. The x-axis represents the slope (r-value) from scores after-drug (post-Edaravone), and the y-axis represents the slope (r-value) from scores before-drug (pre-Edaravone). In each plot, the IDs of participants (subjects) who had sufficient data to compute a slope both before and after starting the drug are highlighted in color. The participants who only had sufficient data to compute slope either before or after drug are represented in a lighter color in the background (their slope is 0 in the other case).

**
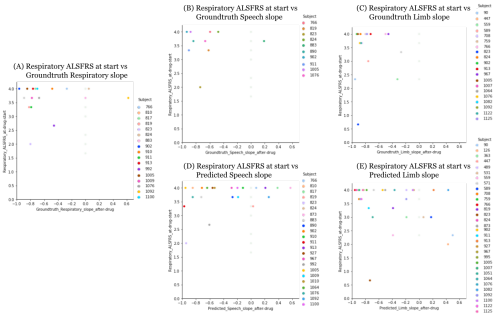
**

**Supplementary Figure 2.** Plots comparing the average respiratory functions’ ALSFRS-R at the start of drug with the slopes (r-values) computed after starting Edavarone on the subset of 54 test participants in the Edaravone cohort. The plots depict respiratory ALSFRS-R (averaged over 3 respiratory-related functions) computed at start or pre-Edaravone and slopes (r-values) computed after (post-Edaravone) using the ALSFRS-R scores: (A) slopes from groundtruth average respiratory scores, (B) slopes from groundtruth speech scores, (C) slopes from groundtruth limb scores [averaged over 6 limb-based movements], (D) slopes from speech scores as predicted by the voice model, and (E) slopes from averaged limb-movement scores as predicted by the accelerometer model. The x-axis represents the slope (r-value) from scores after-drug (post-Edaravone), and the y-axis represents the average respiratory ALSFRS-R at or before starting Edaravone. In each plot, the IDs of participants (subjects) who had sufficient data to compute a slope after starting the drug are highlighted in color. The remaining participants whose data was insufficient to compute slope after drug are represented in a lighter color in the background (their slope is 0).

**
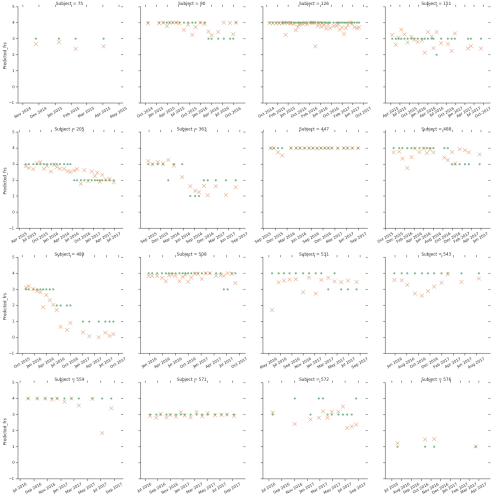
**

**Supplementary Figure 3 A.** [Voice model] 1-of-3 Groundtruth and predicted labels and slopes for before and after drug for all participants in the edaravone cohort. Higher resolution is included in the supplementary materials.

**
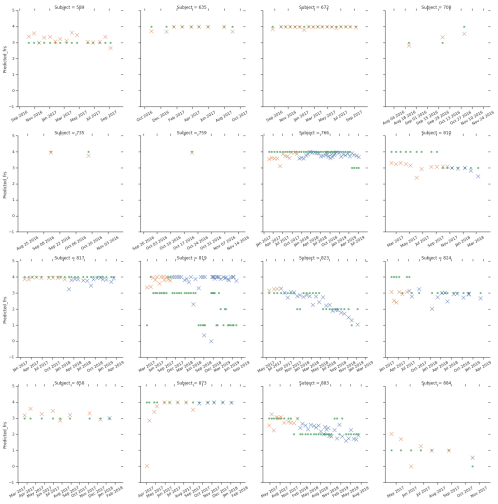
**

**Supplementary Figure 3 B.** [Voice model] 2-of-3 Groundtruth and predicted labels and slopes for before and after drug for all participants in the edaravone cohort. Higher resolution is included in the supplementary materials.

**
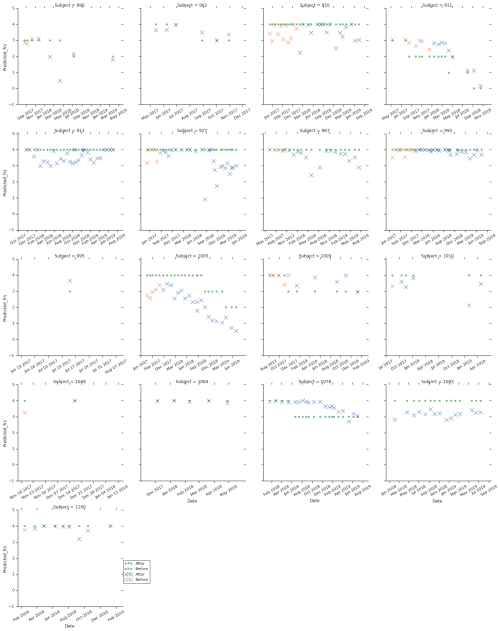
**

**Supplementary Figure 3 C.** [Voice model] 3-of-3 Groundtruth and predicted labels and slopes for before and after drug for all participants in the edaravone cohort. Higher resolution is included in the supplementary materials.

**
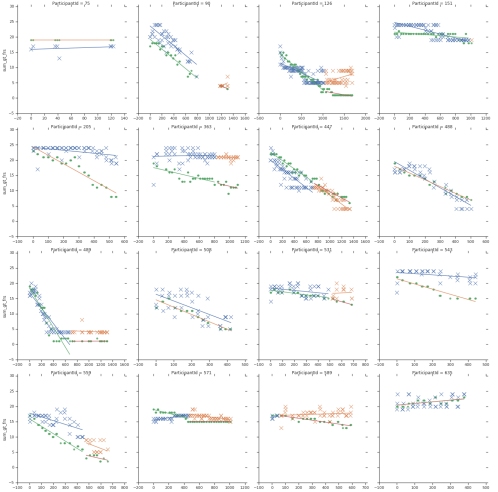
**

**Supplementary Figure 4 A.** [Accelerometer model] 1-of-3 Groundtruth and predicted labels and slopes for before and after drug for all participants in the edaravone cohort. Higher resolution is included in the supplementary materials.

**
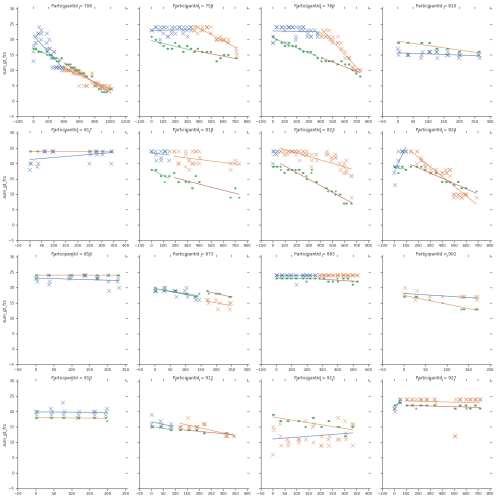
**

**Supplementary Figure 4 B.** [Accelerometer model] 2-of-3 Groundtruth and predicted labels and slopes for before and after drug for all participants in the edaravone cohort. Higher resolution is included in the supplementary materials.

**
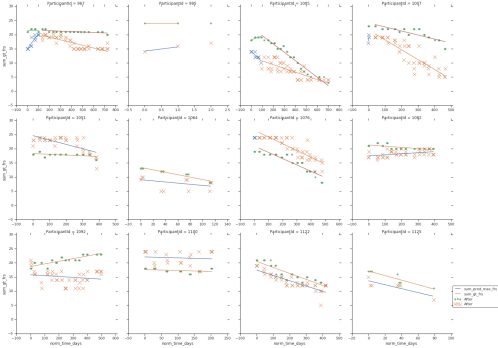
**

**Supplementary Figure 4 C.** [Accelerometer model] 3-of-3 Groundtruth and predicted labels and slopes for before and after drug for all participants in the edaravone cohort. Higher resolution is included in the supplementary materials.
