# Supplementary material for: A machine-learning based objective measure for ALS disease severity
Source: NPJ Digit Med. 2022 Apr 8;5:45. doi: 10.1038/s41746-022-00588-8 (PMC8993812; doi:10.1038/s41746-022-00588-8)
Supplement: Supplementary file 2 — Translational Research Program Clinical Protocol [file 41746_2022_588_MOESM2_ESM.docx]

**STUDY DESCRIPTION – Section A**

| Title of Protocol | ALS Translational Research Program | | |
| --- | --- | --- | --- |
| Principal Investigator | Steven Perrin, PhD | | |
| E-Mail Address | sperrin@als.net | | |
| Co-Investigator | Fernando Vieira M.D. | | |
| E-Mail Address | Fvieira@als.net | | |
| P.I.’s Telephone | 617-441-7236 | P.I.’s Fax | 617-441-7200 |
| Sponsor/Funding Source | ALS Therapy Development Institute (ALS TDI) | | |

#### A1. SIGNIFICANCE AND BACKGROUND FOR THE STUDY

Amyotrophic Lateral Sclerosis (ALS) is a progressive neurodegenerative disorder resulting in progressive muscle weakness, atrophy, and paralysis. ALS is an orphan disease with an incidence rate of approximately 5,000 annual cases diagnosed in the United States on an annual basis. The median survival times after symptom onset is five years resulting a prevalence of the disease in the United States being approximately 30,000 individuals. There is only a single FDA approved treatment for ALS, Rilutek, which offers patients no improvement in quality of life and a minor improvement in survival benefit of 90 days. The development of effective treatments for ALS is a significant unmet need that is hampered by patient heterogeneity. The vast majority of ALS cases are sporadic in nature (90%) with the remaining 10% of genetic cases being associated with over twenty different genes with varying biological functions.

This proposal has several innovative objectives that are critical to developing new treatment strategies, improve clinical trial designs, and develop new quantitative outcome measures for ALS and other chronic neurodegenerative disease conditions.

There have currently been only limited studies that carefully document patient medical history, demographic data and lifestyle with disease progression in ALS. There have been no research or clinical studies that then correlate the clinical phenotype to tools that are now used for drug development and patent stratification such as patient derived induced pluripotent stem cells and whole genome sequencing. Greater than 90% of ALS is sporadic in nature highlighting the need to correlate clinical phenotype and phenotype from iPS cells to background genetics. Therefor this study will also sequence the entire genome from each patient enrolled in the study in order to elucidate genetic background to clinical phenotype.

**Rationale**

In the last decade more than 20 different phase III clinical trials have failed to reach clinical endpoints in ALS clinical trials. Recent advances in genomics and proteomics technologies have opened the door for personalized medicine strategies in clinical oncology. Mutations in v-erb-b2 erythroblastic leukemia viral oncogene homolog 2 (Erbb2, Her-2) and epidermal growth factor receptor (EGFR) have led to genetic screening for these mutations and the development of the FDA approved drugs trastuzumab, Lapatinib, Erlotinib, and Gefitinib.

A critical issue that has hampered the successful advancement of treatments through the clinical trial process in neurodegenerative diseases is patient heterogeneity.

A major advancement that this proposal will create is the software and database infrastructure to link clinical phenotype, genetic background to phenotypic outcomes from cell based assays using iPS cell lines from patients. Patterns from a database of sporadic ALS patients may lead to new targets for drug development in ALS.

# A2. PURPOSE OF PROTOCOL

**Goals of the Proposal**

There are two aims associated with this proposal. The first aim is to characterize the relationships between clinical phenotype through a series of questionnaires, genetic background, representation of phenotype in induced pluripotent stem cells and correlation to drug response in ALS patients. The second aim of this proposal is to identify new end points for ALS clinical trials by comparing the self reported ALS Functional Rating Scale (ALS FRS) to patient voice recordings and data collected from wearable technologies (accelerometers).

In the first phase of the study these goals will be accomplished by obtaining a patient’s consent to collect tissue samples (blood) for DNA sequencing and molecular profiling (RNA and protein) and a skin biopsy to develop an induced pluripotent stem cell line (iPS) for banking and high content drug screening at ALS TDI.

In this phase of the study participants will also be asked to report ALS FRS and provide a voice recording on a monthly basis and to wear accelerometers on their wrists and ankles for one week per month to collect unbiased movement data. These data will be compared to the ALS FRS questionnaire, a self-reporting web based questionnaire to monitor disease progression.

A database will be developed to link a patient’s genetic data, molecular data, and high content screening data with disease progression assessment methodologies.

In the second phase of the study (Extension Program) an additional cohort of early stage ambulatory participants will be recruited to increase the power to detect changes in disease progression using the data from a series of questionnaires, self reported ALS FRS, voice recordings, and wearable technologies (accelerometers).

300 participants will be recruited in the first phase of the study and 700 participants will be recruited in the second phase of the study in order to obtain statistically meaningful data for a total of 1000 participants.

**Specific Aims**

Specific Aim I: Recruit and enroll 300 participants into the phase I part of the study collecting detailed medical records and questionnaires on lifestyle and demographic information.

Specific Aim II: Sequence the genome of each patient entering the study using whole exome and full genome (30X coverage) sequencing technologies.

Specific Aim III: Create Induced Pluripotent Stem Cell Lines from skin biopsies.

Task 1: Reprogram skin fibroblasts from patient biopsies into pluripotent stem cells for banking.

Task 2: Differentiate iPS lines from the skin biopsies of newly diagnosed ALS patients into neurons and astrocytes for phenotypic screens (protein mis-folding, oxidative stress, cell stress) and drug screening assays.

Specific Aim IV: Collect accelerometer data for one week a month to compare the sensitivity of unbiased movement to data to web based ALS FRS self-reporting questionnaire.

Specific Aim V: Collect voice data once a month using an automated system to compare the sensitivity of unbiased movement to data to web based ALS FRS self-reporting questionnaire.

Specific Aim VI: Recruit and enroll 700 additional participants into the phase II part of the study. Collect detailed medical information and questionnaires on lifestyle and demographic information as described in specific aim I. Collect monthly accelerometer data and monthly voice recording data described in specific aims IV and V. Collect quarterly blood samples using an at home venous puncture blood collection. Blood samples maybe used for genome sequencing as described in specific aim II.

#### A3. DESCRIPTION OF RESEARCH PROTOCOL

**A3.1: Overview**

ALS TDI is conducting this study to identify new potential drug targets for ALS and develop new end points for ALS clinical trials. The objectives of this proposal are unique in that it will link clinical phenotype (disease onset and progression over time) with detailed genomic analysis (genome sequence of the participant) and the generation of pluripotent stem cell lines for drug development analysis in the research setting. Blood and skin samples will facilitate these objectives: Blood for DNA sequencing, and molecular analysis, and skin biopsy for generation of pluripotent stem cell lines. The program will assess disease progression by the online collection of data from a self reported ALS Functional rating scale (ALS FRS) questionnaire. The ALS FRS monitors the ability to perform common daily tasks and is used in most ALS clinical trials. Remote voice recording will be collected over the phone to monitor changes in voice patterns over time. Wearable technologies will be used to assess daily activity and exercise compared to ALS FRS.

The proposal will be divided into two phases of participant recruitment.

In phase I of the proposal 300 participants will be recruited over 12 to 18 months to participate in all aspects of the proposal including genomic sequencing, development of iPS cell lines, collection of medical and demographic data, self reported ALS FRS, voice recordings and collection of data from wearable technologies.

In the second phase of the proposal (extension phase) an additional 700 early stage ambulatory participants will be recruited for the collection of medical and demographic data, self reported ALS FRS, voice recordings, and collection of data from wearable technologies. The goal of the extension phase is to increase the power to detect disease progression and compare the gold standard outcome measure to predict disease progression in ALS (ALS FRS) to novel outcome measures (voice recordings, and wearable technologies). Blood samples maybe requested from participants on a quarterly basis during the study for genome sequencing and biomarker studies.

**A3.2: Design and Enrollment Criteria**

**Phase I (300 participants):**

- **Inclusion Criteria**

Participants eligible for inclusion in this study have to fulfill all of the following criteria:

- Male or female healthy volunteers or patients
- Any background ethnicity
- Age 18 years or older.
- Sporadic or familial ALS diagnosed as possible, laboratory-supported probable, probable, or definite as defined by revised El Escorial criteria.
- Capable of providing informed consent.
- Geographically accessible to the site.
- **Exclusion Criteria**

Participants meeting any of the following criteria during screening evaluations will be excluded from entry into the study:

- History or excessive bleeding of on anti-coagulants of any kind including but not limited to daily aspirin, Plavix
- Treatment with an immunosuppressant medication within 30 days of the Baseline visit.
- Active infection (acute or chronic).
- Presence of tracheostomy.
- The presence of unstable psychiatric disease, cognitive impairment, or dementia that would impair ability of the subject to provide informed consent , according to PI judgment, or a history of active substance abuse within the prior year.
- Clinically significant history of unstable or severe cardiac, oncologic, hepatic, or renal disease, or other medically significant illness.

Phase II (Extension study 700 participants)

- **Inclusion Criteria**

Participants eligible for inclusion in this study have to fulfill all of the following criteria:

- Male or female healthy volunteers or patients
- Any background ethnicity
- Age 18 years or older.
- Sporadic or familial ALS diagnosed as possible, laboratory-supported probable, probable, or definite as defined by revised El Escorial criteria.
- Capable of providing informed consent.
- **Exclusion Criteria**

Participants meeting any of the following criteria during screening evaluations will be excluded from entry into the study:

1. Presence of tracheostomy.
2. Symptoms for more than 36 months.
3. The use of non invasive ventilation.
4. The presence of unstable psychiatric disease, cognitive impairment, or dementia that would impair ability of the subject to provide informed consent, according to PI judgment, or a history of active substance abuse within the prior year.
5. Clinically significant history of unstable or severe cardiac, oncologic, hepatic, or renal disease, or other medically significant illness.
6. Participation in the first phase of the study are not eligible to enroll in the extension study.

Patients will be asked to waive their HIPAA rights so that an online database can be developed to track disease progression rate and correlate genetic information from DNA sequencing to phenotypic outcomes.

**A3.3: Description of Study Procedures**

**A3.3.1 Recruitment:**

An email blast will be sent to internal supporters of ALS TDI describing the program after IRB approval. The email blast will be confidential and sent to individual patients registered in ALS TDI’s database. The email will provide a description of the procedures, the aims of the study, a description of the required consent and waivers, and contact information if you are interested in participating in the study. There will also be information on ALS TDI’s website but all members of the ALS TDI community are required to register to utilize these functions on our secure website.

Both of these calls for participation will have a website link to a contact information page. The patient will be asked to enter their name, email address, and phone number. The data will be entered into a database at ALS TDI.

**A3.3.2: Participant Consent**

**Consent: Phase I study participants**

For phase I patients a site visit and travel to Cambridge is required. A clinical coordinator will contact an interested participant by email/phone to set up the prescreening interview. The following information will be provided during the prescreening interview:

- - A clinical coordinator will explain that this is a research study.
  - Participants will be asked to participate in the study for 18 months or until they are no longer able. They can stop at anytime.
  - A clinical coordinator will describe the rationale and purpose behind, the genome sequencing, the development of induced pluripotent stem cell lines, the reporting of monthly ALS FRS, capturing monthly voice recordings, and monthly accelerometer data.
  - There are minimal risks associated with participation in the study.
  - Minor discomfort may be experienced during the venous blood collection.
  - There are no treatments being administered as part of the study.
  - Participation in the study is voluntary and a decision not to participate will not result in any penalty or loss of benefits.

After the pre screening interview if a patient fits the inclusion/exclusion criteria and understands the patient consent requirements and HIPAA requirements for the project they will be enrolled in the study and a clinical appointment set up.

Participants will receive an email. Attached to this email will be a Patient Consent Form & HIPAA form which they can sign electronically using their finger.

Once the participants receive the Patient Consent Form & HIPAA form, study staff will conduct the consenting process over the phone. The participant will not sign the consent until after this occurs.

When the forms arrive back into our system the consent form is then signed electronically by the PI, Steven Perrin.

If a person would like to withdraw or cease to participate they may do so by calling Maeve McNally 617 441 7270, or by emailing either: [mmcnally@als.net](mailto:mmcnally@als.net)

Or [pmphelp@als.net](mailto:pmphelp@als.net)

Participation is voluntary and there is no penalty and no loss of benefits for refusing to participate or withdrawing.

**Consent Phase II study participants**

There is no site visit or clinical visit associated with phase II participants.

If a participant responds to the recruitment announcement they will have provided their name, email address, and phone number.

The system will send a text message to the participant’s phone with a pin number and URL to log into a secure webportal with their preliminary information. The webportal will house all of their data for the study. The text message will serve as an authentication of the identity of the participant.

The webportal will be broken into sections. At the bottom of each section there will be a statement asking if the participant understand all in information provided in that section with a check box to check in order to move on. If a participant has questions an email link and phone number will be provided to contact a study coordinator to clarify information.

The following sections will be broken down:

- - Section I: Overview of the study, its rationale and objectives, and that participants will be asked to participate in the study for 18 months or until they are no longer able. They can stop at anytime.
  - Section II: Describe the rationale and purpose behind, the genome sequencing.
  - Section III: Describe the rationale and purpose for the development of induced pluripotent stem cell lines.
  - Section IV: Describe the rationale and purpose behind collecting monthly self reported ALS FRS.
  - Section VI: Describe the rationale and purpose on collecting monthly voice recordings.
  - Section VII: Describe the rationale and purpose behind collecting monthly accelerometer data.
  - Section VIII: Describe the rationale and purpose behind asking participants to fill out the demographic and medical questionnaires.
  - Section IX: There are minimal risks associated with participation in the study.
  - Minor discomfort may be experienced during the in-home venous blood collection.
  - Section X: There are no treatments being administered as part of the study.
  - Section XI: Participation in the study is voluntary. A decision to not participate will not result in any penalty or loss of benefits.
- Once all the sections have been read and the boxes checked that the participant understands all the information for the study they will be asked to sign the online consent form
- When the forms arrive back into our system the consent form is then signed electronically by the PI, Steven Perrin.
- A PDF version of the consent form will be archived at ALS TDI.
- A printed version of the signed consent will be mailed back to the participant.

**A3.3.3 HIPAA:**

Patients will sign a complete HIPAA waiver allowing ALS TDI to decode their medical history and information and assign these data to all data associated with the project including but not limited to genomic data, molecular profiling data, and data generated from cell lines and tissues. Forms will be archived at ALS TDI a printed version sent back to the participant.

**A3.3.4 Clinical Visit for Participants in Phase I:**

Participants who are interested after the prescreen and have read the waivers and consent will coordinate the visit to ALS TDI through the project coordinator. The project coordinator will contact Mass General Hospital Dermatology Clinic (MGH) and arrange the scheduling for the patient’s visit to MGH for sample collections. MGH is acting as a contract research organization for tissue collection for the project. Subjects will be sent required patient consent and waivers two weeks prior to scheduling their clinical visit. Signed documentation will be verified before scheduling their appointments for skin biopsy, and blood collection.

Patients will be driven to MGH accompanied by an ALS TDI staff person. Copies of all required consents and waivers will be brought to MGH at the time of visit. ALS TDI will provide barcoded sample collection materials for each sample which will contain an anonymous barcode and no patient information. The barcode identifier will link to relevant patient history in ALS TDI’s database. Patient and samples will be delivered back to ALS TDI by an ALS TDI program manager. The skin biopsy will be sent to Charles River by ALS TDI for viral screening and safety then shipped to ALS TDI.

**A3.3.5: Questionnaires for phase I and Phase II participants:**

Once a participant is enrolled and completed their background medical history they will be asked to provide additional lifestyle data at their leisure over the course of the 12 to 18 month study. The list of questionnaires is fixed and could be completed in several hours.

Complete list of Questionnaires

- - - General information
    - Family History
    - Geography
    - Lifestyle
    - Occupation
    - Medical History – Conditions
    - Medical History – Hospital
    - Medical History – Injuries
    - Medications
    - Clinical Trials
    - Vitamins & or Supplements
    - ALS Experience

**A3.3.6: Tissue Collections for Phase I:**

**Blood Draws: There will be a single venous puncture blood draw at the time of the only clinical visit for participants in the phase I study.**

- ALS TDI will provide the phlebotomist at MGH with barcoded PAX gene and heparin collection tubes containing a unique identifier.
- The phlebotomist will collect three tubes for 5 mls of blood into each gene tube for molecular profiling and DNA sequencing

Blood Sample Collection

- Blood collection will be limited to 18 months from the time of initial consent. The investigator and study staff have discretion to determine that an individual should no longer be requested to give blood samples earlier than 18 months if they have concerns that an individual can no longer give continued consent.

**Skin Biopsy Collection: There may be a single skin biopsy at the time of the clinical visit for participants in phase I of the study.**

ALS TDI will provide the dermatologist at MGH with a collection tube containing a unique barcode identifier. The dermatologist will rub numbing cream on the site location for the biopsy, usually from your forearm, thigh or lower back. After the skin is numb, the site will be cleansed with an antiseptic solution and an additional, injectable numbing medication will be given. The injection may sting a little, but the numbing cream should lessen the burning sensation. Once the skin is completely numb, a 3 millimeter piece of skin (about the size of a pencil-end eraser), will be removed. The biopsy site may be closed with a stitch. Participants will be provided with instructions on the care of the skin biopsy site.

**A3.3.7 Tissue Collections Phase II (extension):**

**Blood Collection: At home venous puncture blood collection**

Purpose:

There are no clinical visits scheduled for the extension phase of the study. A blood sample at baseline and at months 3, 6, 9, 12, 15, and 18 will be collected to facilitate the identification of biomarkers that correlate with disease progression outcomes and allow post hoc genomic sequencing as was done for participants in phase I of the study. Participants who are unable to travel to MGH for their initial blood & skin harvest may be invited to provide their samples remotely.

Method:

At home venous puncture blood collection using BD Microtainer tubes butterfly needles and following the manufacturer’s instructions (included below). These blood samples will be used for the isolation of DNA, RNA or blood cells, or for the preparation of plasma and serum samples. Each PMP participant may be asked to provide in-home venous puncture sourced blood quarterly for up to 18 months during their participation in the PMP.

At Home Blood Collection Information

**Prepare Materials for Shipping**

- A blood draw collection kit will be prepared by a clinical coordinator at ALS TDI.
- Up to five uniquely identifiable barcoded blood collection labels will be generated with the patient’s date of birth and 5-digit ID separate to their PMP Patient ID. : PAXgene DNA tube(Product #761115),
- PAXgene RNA tube (Product #762165),
- K2 EDTA tube (367862),
- Plasma preparation tube (Product #362788),
- Serum separator tube (Product #367986), .
- One label will be placed on each appropriate tube.
- A NanoCool Cold Shipping Box (Product #2-85225) will be packed with the aforementioned tubes, multiple pairs of hypo-allergenic nitrile gloves, two biohazard ziplock bags, 2 BD vacutainer safety-lok blood collection sets (REF#367281), 2 sterile isopropyl alcohol prep pads, two sterile gauze sponges (REF#1806), sanitary bench paper, bandage tape, and Therapak foam tube holders for sample tubes once they have been filled with blood.

**Shipping of Materials**

- Two biohazard ziplock bags (REF#DYND30261) will be placed in a NanoCool Cold Shipping Box. One bag will contain the blood tubes and packing foam. The other bag will contain the blood collection sets, alcohol prep pads, sterile gauze sponges, sanitary bench paper, and bandage tape. In addition, the shipping box will contain a prepaid fedex packing slip for return shipping, sample handling instructions, a sticker defining the contents of the box, and Scotch packing tape strips.
- The blood collection protocol will be included in the box.
- The NanoCool Cold Shipping Box will be packaged into an “over-pack”, another cardboard box with its own shipping label. The “over-pack” will be discarded and the NanoCool Cold Shipping Box will be shipped back with the blood samples included.
- An email will be sent to the participant to notify them that the blood collection kit has been shipped via Fedex. The email will also provide a tracking number.

**Preparation for venipuncture blood collection**

- Examination gloves should be worn by the licensed phlebotomist any time the potential for contact with blood exists.  Multiple pairs of nitrile gloves will be provided in the packing kit.
- Open the ziplock back containing the blood collection materials.
- Unfold the sanitary bench paper and place it on an even surface and lay contents of the blood collection kit out.
- Extend the patient’s arm and inspect the antecubital fossa and forearm. Locate a vein of good size that is visible and straight. Vein should be visible without applying the tourniquet.
- Apply tourniquet approximately 4 or 5 finger widths above the venipuncture site.
- Swab the skin where venipuncture will occur with isopropyl alcohol wipes. Allow the alcohol to dry.
- Insert the needle into the vein and collect blood into each tube (K2 EDTA, serum separator tube, plasma separator tube, PAXgene DNA tube, PAXgene RNA tube). A maximum of 2 venipunctures can be attempted per quarterly phlebotomy visit.

**Filling the Collection Tubes**

- Upon filling each tube, invert the tube 8-10 times to activate clotting in the serum separator tube, mix anticoagulant with the blood specimen for K2 EDTA or plasma separator tube, or improve efficiency of cell lysis and DNase/RNase deactivation in the PAXgene tubes..
- An adhesive bandage should then be applied to the punctured vein/skin.

**Shipping Blood Sample to ALS TDI**

- Plasma separator tubes and serum separator tubes will be centrifuged by on-site phlebotomists.
- Following any necessary centrifugation steps, each stoppered blood collection tube should be inserted into a Therapak foam tube holder. All foam encased blood sample tubes should then be placed into the biohazards zip lock bag prior to placing the bag into the NanoCool Cold Shipping Box.
- The NanoCool Cold Shipping Box should then be activated to induce cooling by pressing a button on the center of the underside of the lid of the Styrofoam inner box. The lids should then be placed firmly into the Styrofoam inner box.
- The NanoCool Cold Shipping Box containing blood should be dropped off to the nearest Fedex location by the phlebotomist for return.

Blood Sample Collection

Blood collection will be limited to 18 months from the time of a consented baseline blood draw. The investigator and study staff have discretion to determine that an individual should no longer be requested to give blood samples earlier than 18 months if they have concerns that an individual can no longer give continued consent.

**A3.3.8 Collection of Voice Recordings for Phase I and Phase II Participants**

Voice recordings will be collected in the same fashion in both phase I and phase II of the study.

Voice data will be collected at least once a month using an automated system to compare the sensitivity of unbiased voice recording assessments to web based ALS FRS self-reporting questionnaire.

The Speech Tracking part of the Precision Medicine Program is meant to help add greater sensitivity to interpreting changes in speech by asking participants who are able to record phrases monthly. Use of serial voice recordings was chosen based on research suggesting its ability overtime as a tool to determine changes in a person's ability to speak verbally.

Participants will be required to log into their portal, using their privately protected user name & password. They then provide the telephone number where they can be reached. An automated system will call them within one minute. They would be asked to say any five of the phrases described in Appendix A.

Examples:

- - Who are you talking about?
  - I have no idea.
  - Is she coming?
  - It was nice of you to come today.
  - I don’t remember it being all that hard.

Then hit the # button. The automated system will then thank them and confirm the recording has been made or send them an email if it was not successful.

Alternatively, people could record themselves and send it to me at PMP program Email address pmp@als.net using a MP3 or an Audio recording. I can upload it to their private folder.

Recording:

- Speak in your normal manner
- Start recording
- Pause for 10 seconds at the beginning of the recording.
- Read each phrase aloud as it is presented.
- Pause for 10 seconds at the end of the recording.
- Stop and save your recording
- Send your recording to this email monthly.

Participating in the Voice Recording of the study is completely optional.

Attached please find the screenshots that would guide them through the process.


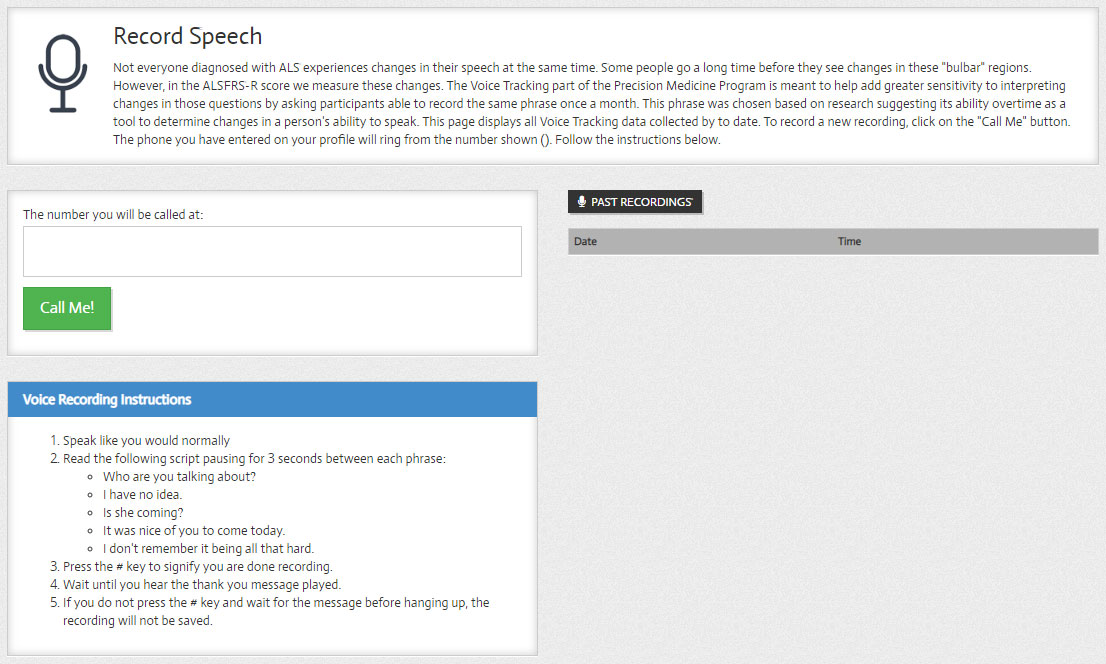


**A3.3.9: Collection of Data from Wearable Technologies (Accelerometers) from Phase I and Phase II Participants**

Collection of data from wearable technologies will be the same for both phase I and phase II of the study.

Accelerometer data will be collected for one week per month to compare the sensitivity of unbiased movement to data to web based ALS FRS self-reporting questionnaire.

ALS is a complex disorder, where the site of onset and progression rate of the disease can vary greatly from person to person. The primary way that the disease is tracked in the clinic is using the ALSFRS-R score, which asks a series of questions about an individual's ability to conduct tasks, such as walking, grooming or feeding oneself. These questions help to asses a person's ability to move their hands, arms, feet and legs; areas often affected first in lumbar onset ALS. However, we believe that we can add greater sensitivity to these measures by asking that Patients to conduct a series of exercises using accelerometers on or around the same time they complete the ALSFRS-R score. Videos of these exercises are available online to help individuals complete the movement protocol.

The movement component of the study invites participants to wear accelerometers on their wrists and on their ankles for 1 week per month. During that week participants would perform a series of movements on three days of the week, whilst seated. They would then send the accelerometers back to ALS TDI in the FED EX Envelope provided. One month later the accelerometers will arrive at their doorstep. They would again wear them for a week and perform the movements for 3 days taking a day of rest between each day of exercise.

Attached is a hyperlink to a video we have created to assist participants in performing the movements. <https://www.youtube.com/watch?v=4G5JaRHxFYM>

We invite participants to participate in this for 18 months or as long the participant desires. If participants desire to withdraw from any elements of the study protocol at any time, they may.

*Step by Step Instructions*

The movements are timed in 45 second increments, and are done in a particular order to isolate motion in each limb one at a time. For the integrity of the data our team will read from your trackers, it is essential that you follow the timing and order of the movements. So please follow “Ken” exactly as he does the movements. Please do all of the motions to the best of your ability. If you experience pain or discomfort while doing any of these motions, either slow down or stop all together and rest out the remainder of the 45 second period. If you cannot do a particular motion to the full extent, please do what you can comfortably. If you cannot do a motion at all, rest during that particular segment.

**After you have identified the Trackers by looking on the back of the device, RW (right wrist), LW (left wrist), RA (right ankle), LA (left ankle) please wear them on the assigned limbs.**

Please make sure the trackers are firmly on your wrists and ankles (but not too tight)

Sit in a comfortable chair with a straight back. It’s ok to sit in a wheelchair.

- To start the exercise set, sit perfectly still for 45 seconds with your arms either at your sides or resting on the arms of the chair. **Get ready to start with your RIGHT LEG exercise in 3, 2, 1**
- (Next), begin your first movement which is to extend your right leg as high as you can and then lower it. Repeat this movement as many times as you can for 45 seconds. Be sure to keep the rest of your body still while you kick your leg. **10 seconds to rest**
- (Then) sit absolutely still for 45 seconds. **Get ready to start with your LEFT LEG exercise in 3, 2, 1**
- (Next) extend your left leg as high as you can, then lower it. Repeat this motion as many times as you can for 45 seconds. Be sure to keep the rest of your body still. **10 seconds to rest**
- Then sit absolutely still for 45 seconds **Get ready to start with your RIGHT ARM exercise in 3, 2, 1**
- (For the third motion), remain seated and swing your right arm out and over your head as high as you can; then lower it. Repeat this movement as many times as you can for 45 seconds. As before, keep the rest of your body still. **10 seconds to rest**
- Then sit absolutely still for 45 seconds. **Get ready to start with your LEFT ARM exercise in 3, 2, 1**
- (For the fourth motion,) remain seated and swing your left arm out and over your head as high as you can; then lower it. Repeat this movement as many times as you can for 45 seconds. **10 seconds to rest**
- Then sit absolutely still for 45 seconds. **Get ready to start with your BEND OVER exercise in 3, 2, 1**
- (For the fifth and final movement), from the seated position, bend over at the waist as if you are going to touch your toes with your hands and then sit back up against the chair. Repeat this movement as many times as you can for 45 seconds. **10 seconds to rest**
- Finally, sit absolutely still for 45 seconds, and then your exercise session is complete.

#### A4. STATISTICAL CONSIDERATIONS

Sample Size Justification: This is a research proposal with no prior knowledge of samples size estimates to determine the number of samples required to correlate genetic background to phenotypic outcome. Given that 90% of ALS cases are sporadic in nature with no known genetic mutation we are assuming it will take greater than 100 established cell lines and genomic sequences to allow un supervised clustering of phenotypic outcomes to genomic sequence. The goal of this phase is to build the tools and data integration systems necessary to perform a first pass analysis of genomics data to clinical phenotype and phenotypic analysis of a patients iPS lines when challenged with exogenous stimuli.

**Rationale around enrolling 700 participants in the second phase and collecting 10,000 data points:**

The most common primary end point in ALS clinical trials is ALS FRS. According to post hoc analysis on dozens of ALS clinical trials it takes 450 patients enrolled in a 12 month study to detect a 30% change in the slope of monthly reported ALS FRS. In order to detect meaningful changes in ALS disease progression slope as small as 20%, a cohort as large as 700 people with ALS would be required. The ALS FRS is a 12 point questionnaire with each question scored from 0-4, 4 being a perfect score. This is then summarized into a single score with a maximal value of 48 points per month for a perfect score. This represents 100,000 data points summarized to 8,400 individual scores over 12 months for 700 patients. In order to compare monthly reported ALS FRS with a potentially new outcome measure such as data from accelerometers the study will require a similar design to compare ALS FRS to accelerometers. The goal of the extension study is to enroll a second cohort of participants, a validation cohort, to collect 15,500 data points for ALS FRS and accelerometers in order to determine the sensitivity of these two potential outcome measures in a statistically meaningful way.


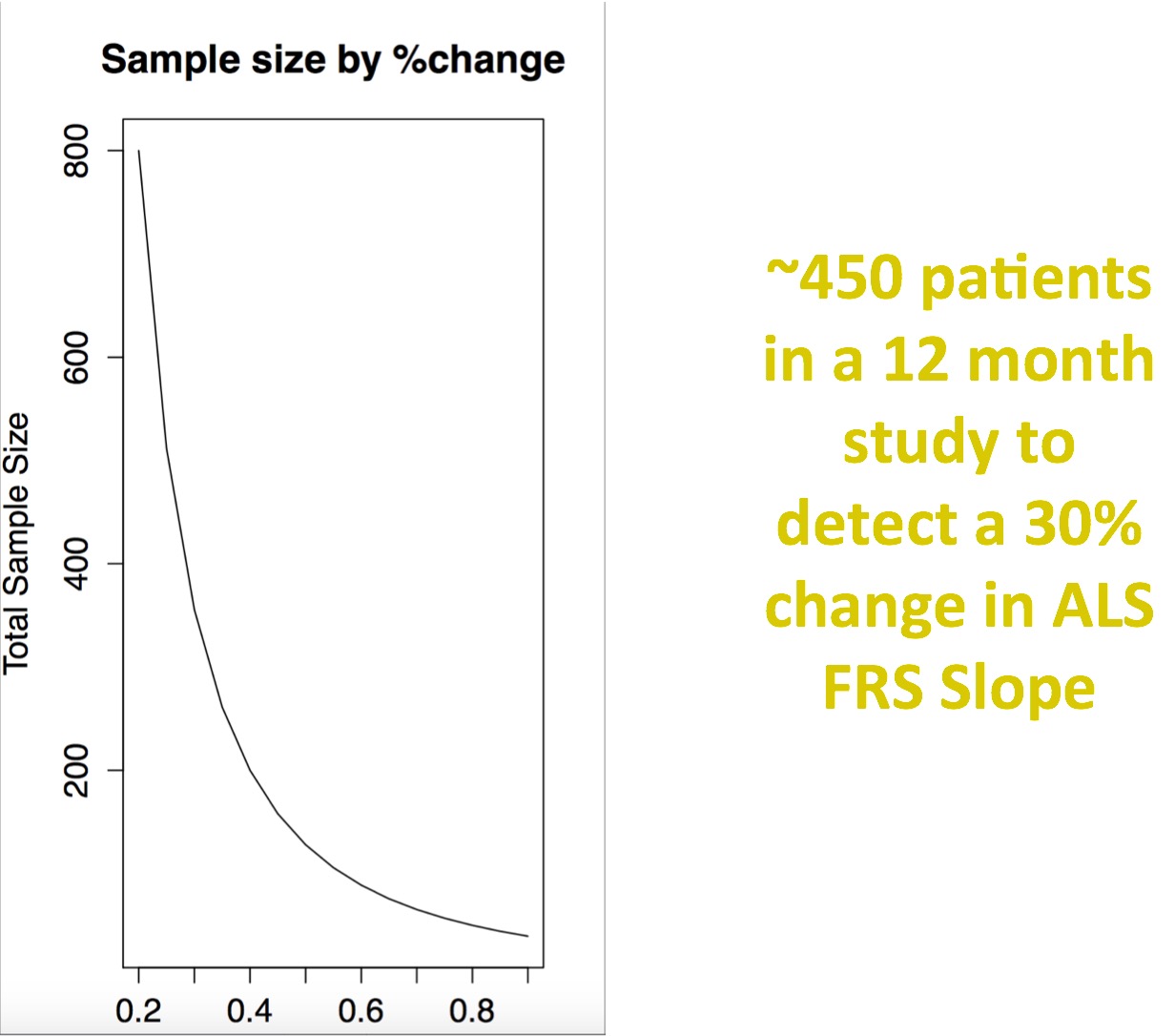


Data Analysis: ALS TDI has a robust data management system that assigns a unique identified and barcode to each tissue sample that is generated from any source. Each unique identifier can then have multiple clinical attributes associated with it including age, gender, age of diagnosis, site of onset, medication history, tissue type etc. ALS TDI has robust genomics capabilities including Affymetrix RNA profiling and whole genome sequencing data infrastructures built in SciTegic Pipeline pilot and a backend using Bioconductor and R code. Data from the project will be entered into these systems for downstream data quality control and processing. The Bioconductor packages have a suite of clustering tools to analyze multidimensional complex data structure to identify patterns in genomics, RNA profiling and clinical data obtained from this project.

The scientific rationale around control group will not be a 1:1 basis but would be of scientifically meaningful controls for example a twin of a participant with spontaneous ALS.

Data Follow Up:

There will be no specified end-time that individuals would be expected to stop reporting data online and the length of their participation in this portion of the research is at their discretion. However if a person would like to withdraw or cease to participate they may do so by calling Maeve McNally 617 441 7270, or by emailing either: [mmcnally@als.net](mailto:mmcnally@als.net)

Or [pmphelp@als.net](mailto:pmphelp@als.net)

Participation is voluntary and there is no penalty and no loss of benefits for refusing to participate or withdrawing.

#### A5. POSSIBLE BENEFITS

There are several beneficial aspects of enrolling in the program. One important aspect is having ones whole genome sequenced as part of the program. The vast majority of ALS cases are sporadic in nature (~90%) with no known genetic association. For patients with the familial form of the disease more than 70% of these cases now have an ability to identify the underlying genetic cause that is associated with about 27 genes. Recent data suggests that it is very common for sporadic cases of ALS to have mutations associated with common ALS genes including SOD1 and C9ORF72. This will allow these patients to know if they have mutations in genes commonly associated with ALS. For sporadic cases with no known mutations the long term goal of clustering the data into clinical phenotypes based on iPS cell phenotypes may allow us to identify new genetic risk factors associated with the disease that maybe relevant to specific ALS cases and their family members.

Another benefit is that the program will bank pluripotent stem cell lines for each patient after reprogramming. From a clinical perspective this technology is still very new and future application to the treatments of patients is quickly evolving. A banked stem cell may prove valuable for a patient as new applications are developed and maybe useful for other family members in the future.

Finally the development of research tools for high content screening from a patient’s own cells could lead to early opportunities for drug repurposing for a patient in the program.

There is a significant benefit for the ALS community as a whole. The construction of a comprehensive database that couples clinical phenotype to genomic sequence data and phenotypic screens on patient cell lines will lead to a more understanding of disease mechanisms, drug development opportunities, and effective treatments for patients. The coupling of genomics to these endpoints will also lead to rare genetic associations with ALS that have not been characterized.

#### A6. POSSIBLE RISKS AND ANALYSIS OF RISK/BENEFIT RATIO

The actual clinical procedures have little risk for patients outside of bleeding from the skin biopsy which in some cases may require a suture to stop the bleeding, and the potential for some scarring around the skin biopsy. There may be localized discomfort from the blood draws.

The most important factor to consider is the need for genetic counseling should a patient choose to want to know any mutations that are identified in the genome sequencing component of the proposal. All ALS clinics have genetic counseling available and for those patients that have a desire to discuss their genetic information ALS TDI will facilitate genetic counseling at their ALS clinical prior to discussions.

With human subjects research, there is always the risk of the loss of confidentiality. ALS will take all reasonable precautions to protect the participant and their data.

#### A7. STUDY LOCATION

All interviews will be conducted over the phone by the project coordinator at ALS TDI.

Patient consent forms and waivers will be distributed to patients and collected via Fedex. The verification of written consent and verbal consent will be conducted at ASL TDI on the day of the visit prior to transport to MGH for tissue collection.

Blood and skin will be collected at MGH Hospital according to the study protocols.

No further patient follow up will be required unless a patient requests to discuss the results of the genetic information.
