# Supplementary material for: A machine-learning based objective measure for ALS disease severity
Source: NPJ Digit Med. 2022 Apr 8;5:45. doi: 10.1038/s41746-022-00588-8 (PMC8993812; doi:10.1038/s41746-022-00588-8)
Supplement: Supplementary file 3 — Reporting Summary [file 41746_2022_588_MOESM3_ESM.pdf]

nature portfolio

Corresponding author(s): Fernando G. Vieira

Last updated by author(s): Jan 20, 2022

# Reporting Summary

Nature Portfolio wishes to improve the reproducibility of the work that we publish. This form provides structure for consistency and transparency in reporting. For further information on Nature Portfolio policies, see our Editorial Policies and the Editorial Policy Checklist.

## Statistics

For all statistical analyses, confirm that the following items are present in the figure legend, table legend, main text, or Methods section.

| n/a | Confirmed | |
|---|---|---|
| ☐ | ☒ | The exact sample size ($n$) for each experimental group/condition, given as a discrete number and unit of measurement |
| ☐ | ☒ | A statement on whether measurements were taken from distinct samples or whether the same sample was measured repeatedly |
| ☒ | ☐ | The statistical test(s) used AND whether they are one- or two-sided<br>*Only common tests should be described solely by name; describe more complex techniques in the Methods section.* |
| ☒ | ☐ | A description of all covariates tested |
| ☒ | ☐ | A description of any assumptions or corrections, such as tests of normality and adjustment for multiple comparisons |
| ☒ | ☐ | A full description of the statistical parameters including central tendency (e.g. means) or other basic estimates (e.g. regression coefficient) AND variation (e.g. standard deviation) or associated estimates of uncertainty (e.g. confidence intervals) |
| ☒ | ☐ | For null hypothesis testing, the test statistic (e.g. $F$, $t$, $r$) with confidence intervals, effect sizes, degrees of freedom and $P$ value noted<br>*Give P values as exact values whenever suitable.* |
| ☒ | ☐ | For Bayesian analysis, information on the choice of priors and Markov chain Monte Carlo settings |
| ☒ | ☐ | For hierarchical and complex designs, identification of the appropriate level for tests and full reporting of outcomes |
| ☒ | ☐ | Estimates of effect sizes (e.g. Cohen's $d$, Pearson's $r$), indicating how they were calculated |

*Our web collection on statistics for biologists contains articles on many of the points above.*

## Software and code

Policy information about availability of computer code

| | |
|---|---|
| Data collection | All computer code were written in python and scripts are available at the following Github. https://github.com/PMPhelp/paper-code |
| Data analysis | All computer code were written in python and scripts are available at the following Github. https://github.com/PMPhelp/paper-code |

For manuscripts utilizing custom algorithms or software that are central to the research but not yet described in published literature, software must be made available to editors and reviewers. We strongly encourage code deposition in a community repository (e.g. GitHub). See the Nature Portfolio guidelines for submitting code & software for further information.

## Data

Policy information about availability of data

All manuscripts must include a data availability statement. This statement should provide the following information, where applicable:

- Accession codes, unique identifiers, or web links for publicly available datasets
- A description of any restrictions on data availability
- For clinical datasets or third party data, please ensure that the statement adheres to our policy

The datasets generated during and/or analysed during the current study are available from the corresponding author on reasonable request.

# Field-specific reporting

Please select the one below that is the best fit for your research. If you are not sure, read the appropriate sections before making your selection.

☒ Life sciences ☐ Behavioural & social sciences ☐ Ecological, evolutionary & environmental sciences

For a reference copy of the document with all sections, see nature.com/documents/nr-reporting-summary-flat.pdf

# Life sciences study design

All studies must disclose on these points even when the disclosure is negative.

| | |
|---|---|
| Sample size | This study was a natural history study enrolling people with ALS gradually over many years. The number of participants involved, 584, was a robust number in order to test whether machine learning tools could be applied to the assessment of ALS disease progression using digital outcome measures. |
| Data exclusions | Data were not excluded. |
| Replication | Measures taken included periodic, up to monthly, self-assessed ALS-Functional Rating Scale(revised) surveys of people living with ALS. In addition, periodic, up to monthly, voice recordings of the phrase: "I owe you a yoyo today." were collected from people with ALS. Finally, periodic, for up to one week out of every month, application of wearable accelerometers were used to collect body movement data from people with ALS. |
| Randomization | All ALS patients meeting enrollment criteria were who consented were allowed to participate in this natural history study. |
| Blinding | There were no placebo controlled experimental assessments involved in this study. |

# Reporting for specific materials, systems and methods

We require information from authors about some types of materials, experimental systems and methods used in many studies. Here, indicate whether each material, system or method listed is relevant to your study. If you are not sure if a list item applies to your research, read the appropriate section before selecting a response.

## Materials & experimental systems

| n/a | Involved in the study | |
|---|---|---|
| ☒ | ☐ | Antibodies |
| ☒ | ☐ | Eukaryotic cell lines |
| ☒ | ☐ | Palaeontology and archaeology |
| ☒ | ☐ | Animals and other organisms |
| ☐ | ☒ | Human research participants |
| ☐ | ☒ | Clinical data |
| ☒ | ☐ | Dual use research of concern |

## Methods

| n/a | Involved in the study | |
|---|---|---|
| ☒ | ☐ | ChIP-seq |
| ☒ | ☐ | Flow cytometry |
| ☒ | ☐ | MRI-based neuroimaging |

## Human research participants

Policy information about studies involving human research participants

| | |
|---|---|
| Population characteristics | All enrollees in this study were men and women with ALS. |
| Recruitment | Participants reached out to the ALS Therapy Development institute |
| Ethics oversight | Advarra IRB reviewed and approved the protocol employed in this study |

Note that full information on the approval of the study protocol must also be provided in the manuscript.

## Clinical data

Policy information about clinical studies

All manuscripts should comply with the ICMJE guidelines for publication of clinical research and a completed CONSORT checklist must be included with all submissions.

| | |
|---|---|
| Clinical trial registration | This study does not prospectively assign participants to any interventions and so is not a clinical trial which requires listing. |
| Study protocol | The full study protocol is available upon request |

| | |
|---|---|
| Data collection | Data were all collected from participants in real-world settings, typically fro their homes. |
| Outcomes | Because there are no prospectively assigned interventions, there were no specific primary or secondary outcome measures. |
